# Using transfixion irrigation with negative pressure drainage (TINPD) minimally invasive to manage infratemporal fossa (ITF) abscess

**DOI:** 10.1097/MD.0000000000033445

**Published:** 2023-05-05

**Authors:** Caiwang Chang, Zhilin Zhou, Mengjia Xie, Juanjuan Gao, Miaomiao Shao, Jinhua Huang, Zhibing Meng

**Affiliations:** a Department of Oral and Maxillofacial Surgery, The Affiliated Hospital of Yangzhou University, Yangzhou University, Yangzhou, Jiangsu, P. R. China; b Dalian Medical University, Dalian, Liaoning, P. R. China.

**Keywords:** infratemporal fossa (ITF), infratemporal space abscess, minimally invasive, negative pressure drainage, odontogenic infection

## Abstract

The treatment of abscess in the infratemporal space is still controversial and bedside and operative intraoral drainage is often used to resolve the abscess. However, it can be difficult to control the infection quickly.^[1]^ In this report, the authors present a new technique of using transfixion irrigation with negative pressure drainage for minimally invasive management of infratemporal fossa abscess.

**Patient concerns:**

A 45-year-old man with type 2 diabetes complained of painful swelling and trismus in the right lower facial region for 10 days. The patient was weak, with mild anxiety, and gradually aggravated.

**Diagnoses:**

The patient was misdiagnosed and received dental pulp treatment for the right mandibular first molar and was given oral cefradine capsules (500 mg 3 times per day). Computed tomography scan and puncture revealed an abscess in the infratemporal fossa.

**Intervention:**

The authors used transfixion irrigation with negative pressure drainage from different directions to reach the abscess cavity. Saline solution was infused through 1 tube and allowed to flow out through the other tube to flush out the pus and debris from the abscess.

**Outcome:**

On day 9, the drainage tube was removed and the patient was discharged. One week later, the patient was followed up in the outpatient clinic and the impacted mandibular third molar was removed. This technique is less invasive and leads to faster recovery times and fewer complications.

**Lessons subsections:**

The report highlights the importance of proper preoperative evaluation, using a thoracic drainage tube as soon as possible, and continuous flushing. A double-lumen drainage tube with a suitable diameter and combined flushing should be designed for future reference. Moreover, the use of drugs can effectively eliminate emboli formation, allowing for faster and more minimally invasive control and removal of the infection.^[2]^

## 1. Introduction

Abscess in the infratemporal space is a rare condition that is usually caused by odontogenic infections, dental extractions, sinusitis, or trauma.^[3]^ It presents with nonspecific symptoms such as trismus and facial pain, and if left untreated, it can spread to multiple spaces and cause severe complications.^[4]^

The infratemporal fossa (ITF) is a complex region that contains important structures such as the mandibular nerve, maxillary artery, pterygoid muscles, and pterygoid venous plexus.^[5]^ It is bordered by various bones and is a challenging region to access and treat infections.

Treatment for infratemporal abscesses is still controversial and there is no consensus on the best approach. In this report, we present a case of a 40-year-old male with an ITF abscess that was treated using transfixion irrigation with negative pressure drainage. This technique involved the use of a thoracic silicone drainage tube and the infusion of saline solution to flush out the pus and debris from the abscess. This method is less invasive than traditional surgical approaches, leads to faster recovery times, and has fewer complications.

## 2. Case report

A 45-year-old man was referred to our department with a complaint of persistent pain and swelling in the right lower facial region, accompanied by trismus, for a duration of 10 days. Initial treatment with dental pulp therapy on the right mandibular first molar and oral administration of cefradine capsules (500 mg, 3 times daily) failed to improve the patient’s symptoms. The swelling in the affected region continued to expand and was accompanied by a constant dull pain that radiated to the right temple. Despite 1 week of intravenous administration of ceftriaxone (500 mg, 3 times daily), the symptoms persisted. The patient reported no history of dyspnea, dysphagia, recent facial trauma, or oral infections. The patient had a history of type 2 diabetes but was not taking any medications. The patient’s family history was unremarkable.

### 2.1. Physical examination

During the extraoral examination, a mild and diffuse elastic soft swelling was observed on the right side of the lower facial region. No signs of redness or swelling were palpable in the cervical, submandibular, submental, or posterior occipital regions. The mouth opening was limited to only 1.5 cm. Upon intraoral examination, the pericoronitis of the right lower third molar was identified, with the gum being swollen and discharging small amounts of pus from the lingual side. Additionally, a fistula was observed at the buccal vestibular sulcus of the right lower first molar. The lower first molar displayed a central opening on the occlusal surface, with percussion pain and tenderness in the apical area.

**Figure 1.**
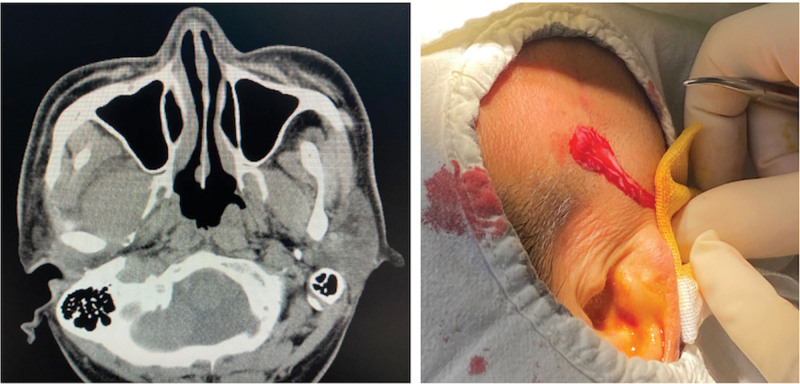
Shows a 1.5 cm × 1.5 cm × 1.0 cm low-density area extending from the pterygomandibular region to the temporal region, suspected to be an abscess with a pus collection in the lateral pterygoid. The incision line was designed on the inferior border of the zygomatic arch, following the direction of the skin. During blunt dissection, yellow purulent secretions were visible in the infratemporal space.

A CT scan showed a 1.5 cm × 1.5 cm × 1.0 cm low-density area ranging from the mandibular condylar medial region to the posterolateral side of the maxilla and pterygoid plate region. This was suspected to be an abscess, indicative of a collection of pus in the ITF (Fig. 1). Blood test results showed elevated leukocytes (13,560/μL) and c-reactive protein (186.22mg/dL). Biochemical examination revealed a glucose level of 9.85 mmol/L, a 2-hour postprandial blood glucose level of 13.07 mmol/L, a glycosylated hemoglobin (A1c) of 8.5%, a serum sodium level of 128.5 mmol/L, and a serum chloride concentration of 92.1 mmol/L.

**Figure 2.**
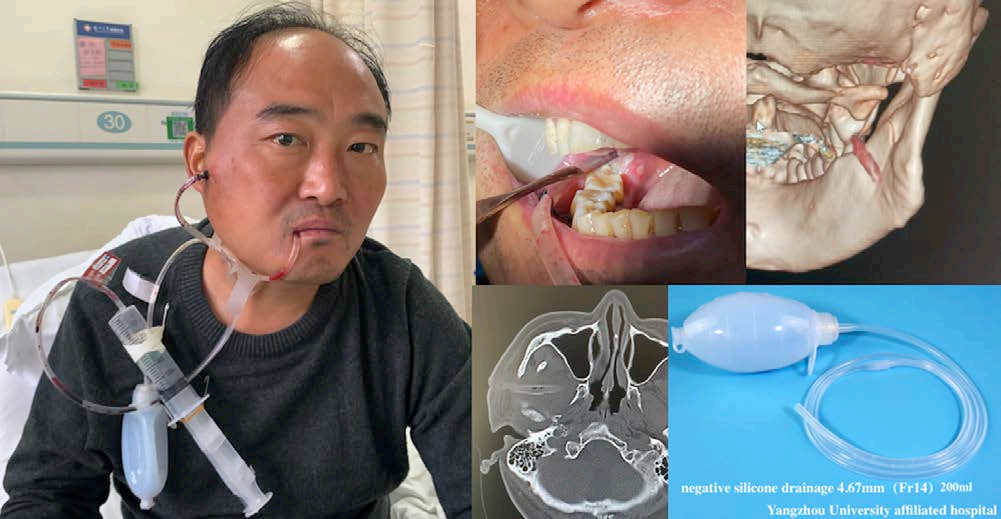
Shows the placement of the drainage tube in the right pterygoid-mandibular space and infratemporal space on day 1.

**Figure 3.**
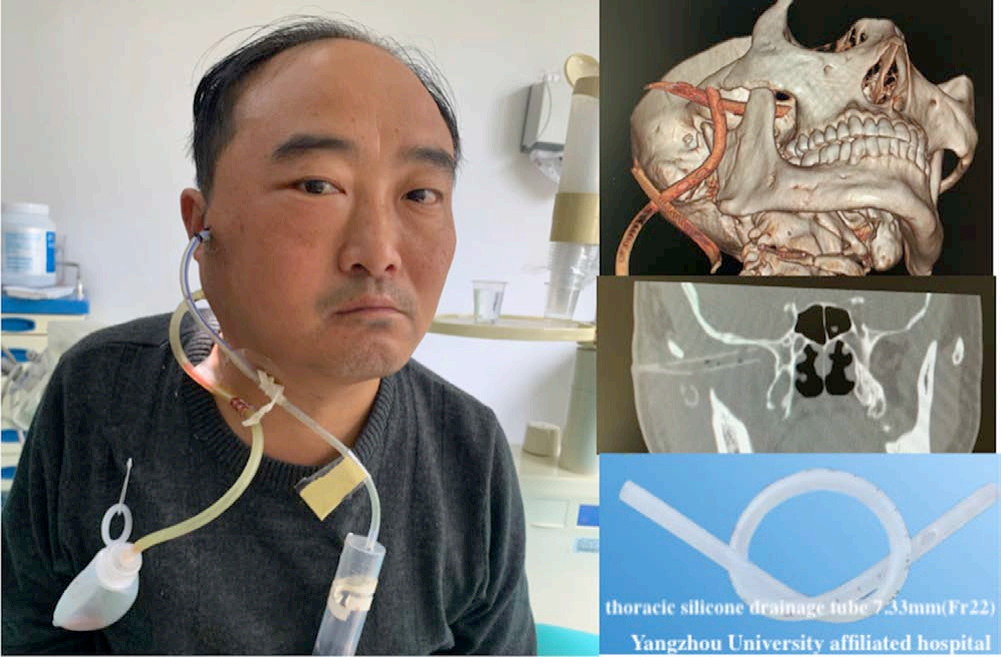
Shows that the right facial swelling worsened, spreading to the temporal and parotideomasseteric regions, with limited mouth opening and numbness in the upper lip and infraorbital region. The drainage tubes were inserted into the lateral border of the mandibular ramus and then connected to the temporal drainage tube at the mandibular notch.

**Figure 4.**
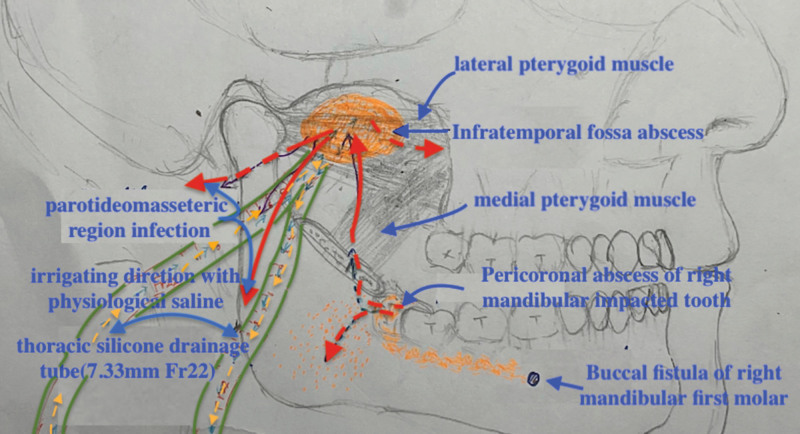
The schematic diagram illustrates that LR8 of peri coronal abscess resulted in infection of the pterygoid-mandibular space, LR6 of buccal fistula, with the upward formation of infratemporal space infection and diffusion to the parotid masseter muscle area and the pterygoid maxillary fissure space.

**Figure 5.**
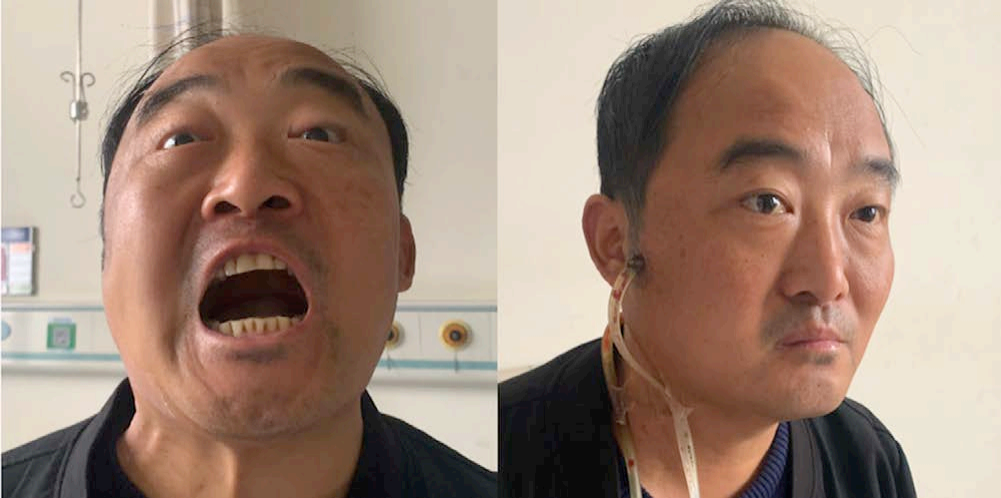
Showed that after the drainage was clear and without purulent secretions, it was removed. The improvement in swelling and restricted mouth opening was significant.

The patient was hospitalized for treatment of difficult food intake and painful swelling caused by an abscess in the ITF. Drainage treatment was performed with an incision made at the buccal skin below the zygomatic arch, avoiding the zygomatic branch of the facial nerve. A drainage tube (4.67 mm Fr14, Fig. 2) was placed in the right pterygoid-mandibular and infratemporal spaces. Mezlocillin sodium and sulbactam sodium3.75 g (q8 hours) with metrogyl (0.5g bid) was administered intravenously for 2 days. The oral negative drainage tube was removed after 2 days due to reduced oral drainage. On the third day, the patient’s right facial swelling and pain worsened, radiating to the temporal region, with limited mouth opening and an increased fever of up to 39.4 degrees. Upon examination, upper lip and infraorbital numbness, and soft tissue swelling were observed on the lateral aspect of the mandibular ramus. During puncture, approximately 2 mL of yellow pus was collected at the posterior border of the mandibular ramus. Under local anesthesia, the temporal drainage tube was adjusted and replaced with a thoracic silicone drainage tube (7.33 mm Fr22) with the removal of a residual embolus from necrotic tissue. The tube at the mandibular angle was then connected to the temporal drainage tube at the mandibular notch. (Fig. 3) The patient was given a 250 mL rinse with physiological saline twice daily and the antibiotic was upgraded to intravenous Imipenem and Cilastatin Sodium for Injection (1g q8 hours) (Fig. 4). Pus cultures showed negative results for both anaerobic and aerobic bacteria. From day 4, the patient’s fever stopped and the pain disappeared, allowing for normal sleep. C-reactive protein significantly decreased on day 5 and returned to normal on day 7. While fasting blood sugar had fluctuations, it was generally controlled below 8 mmol/L with the use of Pioglitazone and Metformin (oral route 15 mg bid). On day 9, the drainage was clear and free of purulent secretions (Fig. 5) and a review of the blood routine was normal. The drainage tube was removed, the antibiotic infusion was stopped, and the patient was discharged after observation for 1 day. One week later, the patient was followed up in the outpatient clinic and an impacted mandibular third molar was removed.

## 3. Discussion

ITF infection is an acute, suppurative inflammation of the infratemporal space that can result from the spread of bacteria from adjacent spaces such as the pterygomandibular space or from maxillary tuberosity, foramen ovale, or foramen rotundum block anesthesia. It can also occur due to periapical infection of maxillary molars or after tooth extraction.^[6]^ In this case, pericoronitis of the right lower third molar has spread the bacteria to the pterygomandibular space, as there are sparse connective tissue spaces between the mandibular coronoid process and the posterolateral maxilla that allow for the spread of the infection into the ITF. Uncontrolled diabetes exacerbates the process of infection and can lead to local necrotizing fasciitis and abscess formation.^[7]^

The position of the ITF is located in a deep and hidden area, making it difficult to identify signs of infection. Upon careful examination, swelling in the upper and lower zygomatic arch and the back of the mandibular ramus may be observed, along with tenderness and limited mouth opening to varying degrees. As the infection progresses, the affected area may experience decreased swelling, tenderness, and pain while chewing, leading to related symptoms if important anatomical structures are impacted.^[8]^

Initially, the patient was misdiagnosed with periapical inflammation of the right mandibular first molar due to restricted mouth opening and pain on the right side of the face, leading to a pulp treatment being performed. Later physical examination only revealed pericoronitis and an abscess of a wisdom tooth, with no apparent swelling on the face.^[9]^ However, white blood cell count and C-reactive protein examination, computed tomography scan, and puncture showed that an abscess had formed in the ITF. Such cases are often difficult to diagnose in clinical practice due to lack of experience, leading to misdiagnosis or missed diagnosis, including cases where patients are diagnosed with the temporomandibular joint disorder, causing increased patient suffering and delayed treatment.^[1,4]^

In this case, the patient developed an infection centered in the infratemporal space under uncontrolled diabetes mellitus. A negative pressure drainage tube was placed between the mandibular notch and the inferior middle border of the zygomatic arch, along the lateral pterygoid muscle to the ITF, and another tube was placed in the pterygomandibular space. Empirical antibiotic therapy was initiated to prevent the infection from worsening, but the patient’s condition continued to deteriorate. The infection spread to the parotid masseteric and temporal regions and even affected the trigeminal nerve,^[10]^ leading to numbness of the upper lip, which indicated a spread to the pterygomaxillary fissure. The patient was at risk of developing intracranial and ocular infections due to spread through the venous plexus and ophthalmic vein.^[11]^ No bacterial growth was observed in the 2 pus cultures, possibly due to previous treatment with intravenous antibiotics. Streptococcus species are still the most common causative pathogen in patients with or without diabetes mellitus.^[12]^ The treatment was upgraded with empirical antibiotics and hyperglycemia control. Efforts were also made to effectively drain and remove abscesses and necrotic tissues by repeatedly irrigating the right masseter area and connecting it to the ITF to promote pus and necrotic tissue discharge.

In the treatment of maxillofacial infections, a conventional negative pressure drainage tube is often used. However, it has been noted that the tube can become obstructed by emboli formed from decomposed necrotic tissue and fluid exudates, leading to ineffective control of the infection. To address this issue, we use a thoracic FR22 silicone catheter. The catheter has a firm texture, which makes it less prone to collapse, and is twice the diameter of the conventional drainage tube. This allows for effective drainage of viscous pus in maxillofacial infections, reducing the risk of congestion. Literature review has shown that the use of negative pressure drainage with irrigation technology in the treatment of maxillofacial infections has been shown to accelerate the drainage of pus and prevent blockage of the drainage tube.^[13,14]^ There are also double cavity drainage tubes with built-in irrigation and continuous irrigation available, which can effectively shorten the treatment time and reduce patient discomfort.^[2]^ In this case, it was found that the patient had not only an abscess but also fascial tissue necrosis. Traditional surgical methods such as large-scale incision and drainage could not be performed due to anatomical limitations. Negative pressure drainage and irrigation, however, were able to effectively remove the abscess and necrotic tissue. Although an Fr22 drainage tube was used during the treatment, embolus formation was observed in the drainage tube cavity 2 days later, reducing the effectiveness of irrigation and drainage. After prompt cleaning, the irrigation and drainage process continued smoothly, leading to a rapid improvement in the patient’s symptoms, including reduced fever, pain, and improved mouth opening. However, the patient’s upper lip numbness persisted at discharge.

## 4. Conclusion

ITF infections are a rare and potentially life-threatening complication of odontogenic infections. In this case, physical findings may remain hidden until the infection develops into an ITF abscess. An accurate understanding of the extent of the infection and active use of antibiotics is important, but the key factor is to achieve sufficient drainage as soon as possible. In this case, we used a large-diameter drainage tube with penetrating irrigation and cleaned up the embolus in the tube in a timely manner. The patient received ideal treatment in the short term and this approach is worth promoting for similar infections.

## Author contributions

Investigation: Caiwang Chang, Miaomiao Shao, Juanjuan Gao.

Methodology: Caiwang Chang, Juanjuan Gao, Jinhua Huang.

Resources: Caiwang Chang.

Supervision: Zhibing Meng.

Software: Mengjia Xie, Juanjuan Gao.

Writing – original draft: Caiwang Chang, Zhilin Zhou.

Writing – review & editing: Caiwang Chang.
